# Supplementary figures and images for: Pushing the Boundaries: Forensic DNA Phenotyping Challenged by Single-Cell Sequencing
Source: Genes (Basel). 2021 Aug 30;12(9):1362. doi: 10.3390/genes12091362 (PMC8466929; doi:10.3390/genes12091362)

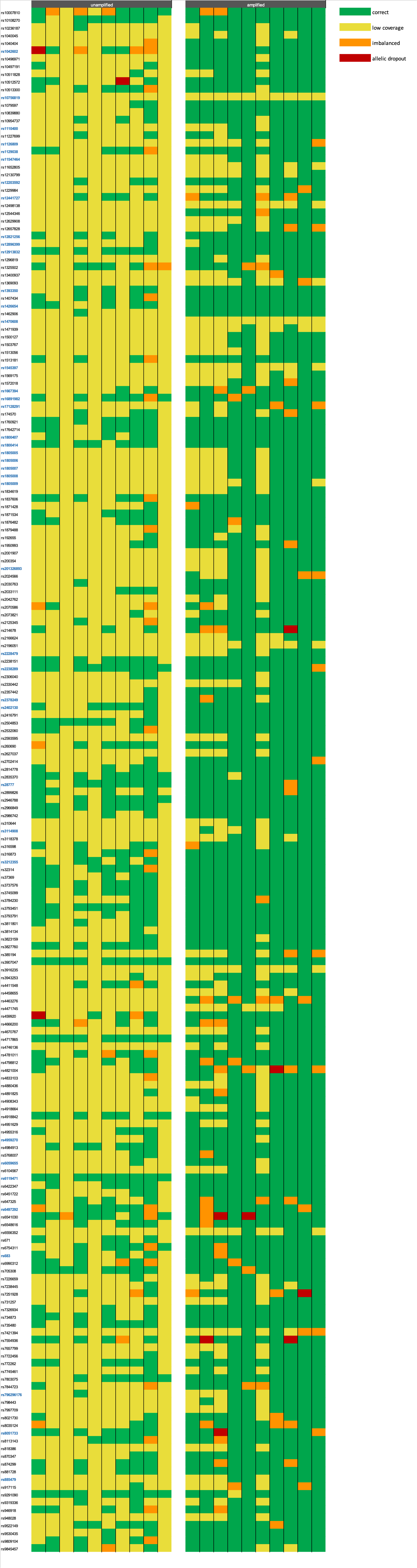

Supplement: Supplementary file 1 [file genes-12-01362-s001.zip › suppl/Figure S2.png]

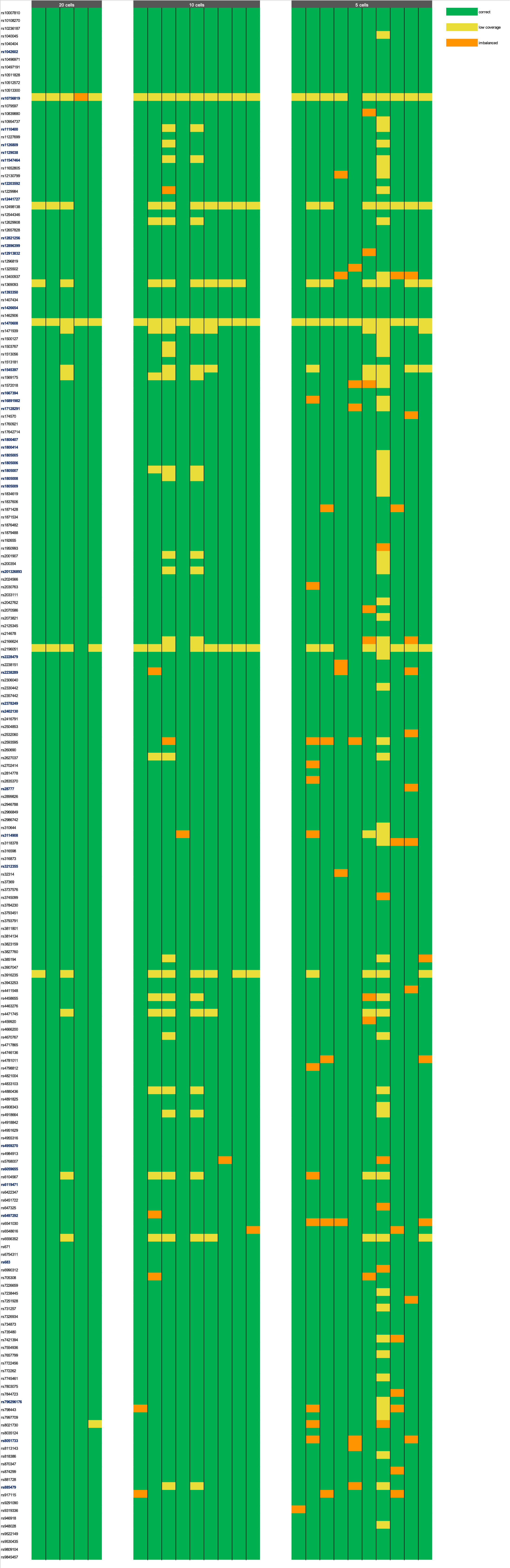

Supplement: Supplementary file 1 [file genes-12-01362-s001.zip › suppl/Figure S1.png]
